# Comparison of Outcomes among Chronic Kidney Disease V Patients with COVID-19 at the National Kidney and Transplant Institute: A Retrospective Cohort Study

**DOI:** 10.1155/2022/1148378

**Published:** 2022-01-06

**Authors:** Maria Fe Bautista, Romina Danguilan, Mel-Hatra Arakama, Roxan Perez

**Affiliations:** ^1^Division of Adult Nephrology, National Kidney and Transplant Institute, Quezon City, Philippines; ^2^Division of Internal Medicine Section of Hematology, National Kidney and Transplant Institute, Quezon City, Philippines

## Abstract

**Background:**

There is very little published data on outcomes of COVID-19 among chronic kidney disease (CKD) patients. We compared the outcomes of COVID-19 in a tertiary care renal hospital among CKD V patients on hemodialysis (HD), peritoneal dialysis (PD), and dialysis initiation, in terms of duration of hospitalization, in-patient mortality, and 30-day mortality.

**Methods:**

A total of 436 CKD V patients, on either HD, PD, or dialysis initiation, with COVID-19 who were admitted at the National Kidney and Transplant Institute (NKTI) from March 13, 2020, to August 31, 2020, were included. Kaplan–Meier survival analysis was performed. Comparison of probability of mortality by group was performed using Log-Rank test. *p* values ≤0.05 were considered statistically significant.

**Results:**

Among 436 CKD V patients, 298 (68%) were on HD, 103 (24%) were on PD, and 35 (8%) required dialysis initiation. Overall in-hospital mortality was 34%; 38% were on HD, 20% on PD, and 37% on dialysis initiation. Total 30-day mortality was 27%; 32% were on HD, 26% on PD, and 16% on dialysis initiation. Median follow-up was 24 days. Among the 137 deaths recorded, total median time to death was 10 days; 8.5 days, 15.5 days, and 9 days for HD, PD, and dialysis initiation groups, respectively. Probability of mortality was significantly higher in HD patients versus PD patients (*p* < 0.00001) and in the dialysis initiation group compared to PD patients (*p*=0.0234). Mortality probability, however, was not significantly different in HD patients versus the dialysis initiation group (*p*=0.63).

**Conclusion:**

Among CKD V patients diagnosed with COVID-19 at the NKTI, those on HD and on dialysis initiation had significantly higher in-hospital and 30-day mortality, compared to patients on PD.

## 1. Introduction

The pandemic of COVID-19 caused by severe acute respiratory syndrome coronavirus 2 (SARS-CoV-2) spread to the Philippines on January 30, 2020, when the first case was confirmed in Metro Manila [1]. The first local case was confirmed on March 5, 2020 [2].

Various studies have described the clinical characteristics and outcomes of CKD patients with COVID-19. Guan et al. reported in China that among patients with COVID-19 with serum creatinine ≥133 *μ*mol/liter, 4.3% had severe disease and 9.6% reached the primary endpoint of admission to the intensive care unit (ICU), use of mechanical ventilation, or death [3].

In another single-center study by Yiqiong et al. in Wuhan, 42 cases (18.26%) of COVID-19 developed in 230 HD patients. Symptoms were mild in the majority, with none admitted to the ICU. Ten infected patients died. This study reported that HD patients with COVID-19 were likely to experience mild disease probably due to reduced function of the immune system and decreased incidence of cytokine storm [4].

In reports from 2 hospitals in Wuhan, prevalence of COVID-19 among dialysis patients was 16% (137 of 857), and mortality rate was 13.1% (18 of 137) [4, 5]. Among those who died, symptoms were less aggressive; thus, it was posited that dialysis patients may be relatively protected from the violent cytokine storm due to their impaired immune system [5].

These Asian studies were in contrast with a Spanish study analyzing the clinical course and outcomes of 36 hospitalized HD patients with COVID-19. They found a higher mortality rate at 30.5% (11 of 36). Factors associated with mortality were longer dialysis vintage, increased lactate dehydrogenase (LDH), increased C-reactive protein (CRP) level, and decreased lymphocyte count [6].

An Italian study also found a high mortality rate of 29% among 94 HD patients with COVID-19. Mortality rate among the admitted was higher at 42% (24 of 57). Risk factors for death included fever, cough, and elevated CRP [7].

Conversely, as PD patients comprise only 11% of the global dialysis population, there is paucity of data on PD patients with COVID-19 [8]. PD patients are also considered immunocompromised, which may be associated with high morbidity of infection [9]. Data are limited and, in 2 case reports, 1 with atypical presentation of nausea and vomiting and the other a case of isolation of the virus in the dialysate fluid, both patients were admitted and discharged recovered [10, 11].

In this study, outcomes among CKD V patients with COVID-19 on HD, PD, or requiring dialysis initiation will be compared.

By comparing outcomes among CKD V patients who had COVID-19, this study can identify factors that can be modified and measures that can be instituted to prevent poorer outcomes associated with this immunocompromised population and, hence, help improve survival.

## 2. Methods

This is a single-center retrospective study. The primary objective of this study was to compare outcomes among CKD V patients infected with COVID-19 in terms of duration of hospitalization, in-patient mortality, and 30-day mortality. Secondary objectives were to determine differences and similarities in the characteristics of CKD V patients requiring dialysis initiation on HD and on PD diagnosed with COVID-19 according to (1) baseline characteristics, laboratory and radiologic features, (2) mean net fluid removal by week, and (3) treatment administered for COVID-19.

Included cases were CKD V patients requiring dialysis initiation on HD or on PD admitted at the NKTI; at least 19 years old; diagnosed with COVID-19 using the oropharyngeal/nasopharyngeal swab real-time polymerase chain reaction (OPS/NPS RT-PCR) as the gold standard within the study period of March 13 to August 31, 2020. Patients meeting the following criteria were excluded: (1) requiring acute dialysis or who have been on HD or PD for less than 90 days, (2) with a failed kidney transplant graft, and (3) with congestive heart failure class IV, chronic obstructive pulmonary disease requiring oxygen, liver cirrhosis, malignancy, or peripheral vascular disease with at least below-the-knee amputation.

### 2.1. Definition of Terms

- Chronic kidney disease V: presence of kidney damage or decreased kidney function for ≥3 months irrespective of the cause, established via ultrasound, staged via glomerular filtration rate of <15 ml/min/1.73 m2.
- Hemodialysis: type of renal replacement therapy where a dialyzer and a dialysis machine are used for removal of metabolic waste products and fluid
- Peritoneal dialysis: type of renal replacement therapy wherein metabolic waste products and fluid are removed by infusion of a dextrose-containing solution into the peritoneal cavity and allowing it to dwell for a set period of time
- Dialysis initiation: CKD V patient not on HD or PD, or with a failed kidney transplant graft on admission
- Charlson comorbidity index: scoring system predicting 10-year survival in patients with multiple comorbidities
- In-hospital mortality: death occurring during the hospitalization
- 30-day mortality: death occurring within 30 days from date of admission

### 2.2. Sample Size

All CKD V patients with COVID-19 from March 13, 2020, to August 31, 2020, were included.

Using Epi Info 7, sample size was computed using 95% confidence level, 80% power, 29% mortality among HD and 2 times the risk of mortality in CKD patients on PD from the study of Alberici, et al.[7]. The minimum sample size required for this study is 429 CKD V COVID-19 patients. A total of 436 patients were included.

### 2.3. Data Collection Procedure

This research passed through the technical and ethical review and approval of the NKTI ethics committee. Approval to collect and use medical records of COVID-19 patients was sought from the medical records head. Information on the following was collected: age, sex, primary kidney disease, comorbidities, dialysis modality, duration of dialysis, mean net fluid removal, laboratory and radiologic features, treatment, and outcomes. The 30-day outcome was gathered through records of the attending physician. Loss to follow-up and missing outcome data were recorded.

Data collected were encoded in Microsoft Excel and were checked and validated to correct encoding errors or missing data, if any. Encoded data was checked for consistency, ranges, and validity. Data privacy was adhered to in terms of access, storage, and disposal.

### 2.4. Statistical Analysis Plan

Stata MP version 14 software was used for data processing and analysis. Continuous data were presented as median/interquartile range (IQR) and analyzed using Kruskal–Wallis test or Mann–Whitney *U* test. Significant Kruskal–Wallis test was further analyzed using Dunn's test. Categorical data were presented as frequency/percentages and were analyzed using Fisher's exact test or chi-square test. Kaplan–Meier survival analysis was performed, in which Time 0 was the time of hospital admission. Comparison of probability of mortality by group was performed using Log-Rank test. *p* values ≤0.05 were considered statistically significant.

### 2.5. Ethical Considerations

The protocol of this study adhered to the ethical considerations and ethical principles set out in relevant guidelines, including the Declaration of Helsinki, WHO guidelines, International Conference on Harmonization-Good Clinical Practice (GCP), and National Ethics Guidelines for Health Research.

The study only commenced upon the approval of the Institutional Review Board of the National Kidney and Transplant Institute.

Subject information was kept in a computerized Excel format file and stored on a personal computer with password access. A code number was assigned to each patient to maintain anonymity. Only the researchers and members of the NKTI REC (Research Ethics Committee) will have access to records.

The investigators have completed the GCP training on responsible conduct of research with human data.

## 3. Results

Among 436 patients included in the study, 298 (68%) were on HD, 103 (24%) were on PD, and 35 (8%) were on dialysis initiation. Median age was 52.5, ranging from 19 to 92 years old. Majority were <60 years old. There was no significant difference in median age and age category by group. A slightly higher proportion of patients were males. There was no significant difference across the 3 groups in terms of sex (Table 1).

The most common cause of primary kidney disease was diabetes mellitus (DM) (33%). There was no significant difference across the 3 groups in terms of DM, hypertension, and chronic glomerulonephritis (CGN). However, a significantly higher proportion of patients requiring dialysis initiation had other causes of primary kidney disease compared to HD and PD.

Median Charlson comorbidity index was 4, ranging from 2 to 10. There was no significant difference across the 3 groups based on Charlson comorbidity index.

Among all the symptoms listed, difficulty in breathing was the most common chief complaint. A significantly higher proportion of HD patients reported difficulty of breathing (*p* value 0.0007) compared to PD and dialysis initiation patients. A significantly higher proportion of PD patients had loss of smell/taste (*p* value <0.0001).

Among 401 patients who underwent either HD or PD, median dialysis duration was 1 year. There was no significant difference in median dialysis duration between HD and PD patients.

Median net weekly fluid removal was 5376.50 ml. Median net weekly fluid removal significantly differed by group. Further analysis using Dunn's test (data not shown in table) showed that median net fluid removal in the HD group was significantly higher compared to the PD group (*p* < 0.00001) and was significantly lower than the dialysis initiation group (*p*=0.0004).

As shown in Table 2, baseline laboratory values among the 3 groups were mostly normal, except for albumin, which was significantly lower in PD compared to HD (*p* < 0.00001) and dialysis initiation (*p*=0.0001) patients. Hemoglobin, platelet, sodium, and potassium, though with significant difference among the groups, were all normal.

Inflammatory markers (Table 2) including LDH, hs-CRP, and procalcitonin were all significantly higher in the HD group compared to PD group (*p*=0.0307, *p*=0.0299, *p*=0.0008, respectively). LDH and procalcitonin were both significantly higher in the HD group compared to dialysis initiation group (*p*=0.0171, *p*=0.0188, respectively).

Baseline radiologic features were also compared. About half of the patients had multilobar pneumonia. However, no significant difference in radiologic features was observed across the 3 groups.

Hemoperfusion was the most common (10%) treatment given. The proportion of patients who received each treatment listed in Table 3 did not significantly differ across the 3 groups, except for tocilizumab.

As seen in Table 4, in-hospital mortality was 34% (147 of 436) and was found to be significantly different among the 3 groups. It was highest in the HD group at 38% (113 of 298), followed by the dialysis initiation group at 37% (13 of 35), and lowest in the PD group at 20% (21 of 103). Thirty-day mortality was 27% (119 of 436) and was also found to be statistically different among the groups. Similarly, it was highest in the HD group at 32% (94 of 298), followed by the dialysis initiation group at 26% (9 of 35), and lowest in the PD group at 16% (16 of 103).

For the succeeding analysis, 18 patients (17 expired, 1 discharged on same day of admission) were excluded. Four hundred eighteen (418) patients were included. Median follow-up time was 24 days [IQR: 8–71, range: 1–221 days].

**Figure 1:**
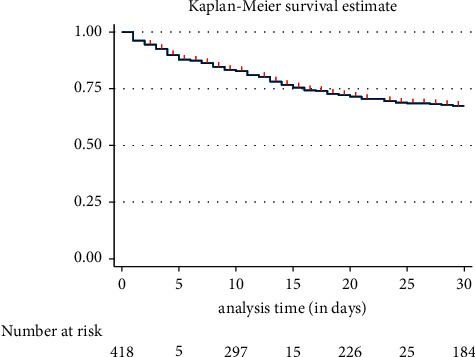
Kaplan–Meier graph showing the probability of survival of CKD V patients with COVID-19 (*n* = 418).

Figure 1 shows that 30-day mortality incidence was 32.51% (95% CI:27.89–37.68%). Of 119 deaths within 30 days recorded, median time to death was 8 days [IQR: 3–14 days; range: 1–29 days].

**Figure 2:**
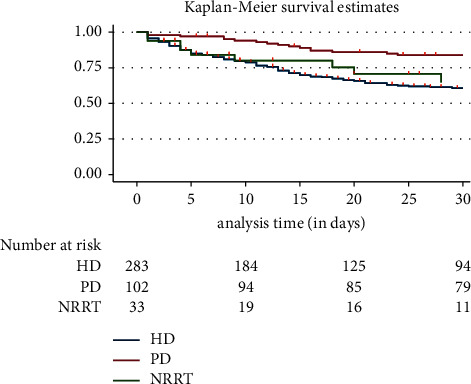
Kaplan–Meier graph showing the probability of survival of CKD V patients with COVID-19 by group (*n* = 418).

Figure 2 shows that 30-day incidence of mortality was 39.29% (95% CI: 33.22–46.04%), 16.04% (95% CI: 10.15–24.85%), and 35.22% (95% CI: 19.44–58.18%) of patients in the HD, PD, and dialysis initiation groups, respectively.

Median time to death among the 94 HD patients was 7 days [IQR: 3–14, range: 1–29 days], 16 PD patients 12.5 days [IQR: 8–16, range: 1–24 days], and 9 dialysis initiation patients 5 days [IQR: 4–18, range: 1–28 days].

Probability of mortality was also compared by group. Log-Rank test was significant (*p*=0.0001). Pairwise analysis revealed that probability of mortality was significantly higher in the dialysis initiation versus the PD group (*p*=0.0234) and was significantly higher in the HD versus PD group (*p* < 0.00001). Conversely, there was no significant difference in probability of mortality in the HD versus dialysis initiation group (*p*=0.6300).

## 4. Discussion

Most of the CKD V patients with COVID-19 admitted at the NKTI for the study period were on HD, reflecting that majority of this special population in the Philippines is on HD. Baseline characteristics across the HD, PD, and dialysis initiation groups with COVID-19 were comparable. Majority were <60 years old, with DM as their primary kidney disease. Median Charlson comorbidity index was 4 for all groups, signifying a moderate risk for mortality from comorbid diseases, or a 53% estimated 10-year survival for all, which may be related to a large number of them having both CKD and DM as comorbidities [12].

In terms of chief complaint, difficulty of breathing was the most common in all 3 groups, which is one of the usual presenting symptoms of COVID-19 [13]. However, it was significantly higher in the HD group, while loss of smell/taste was significantly higher among those on PD. This was similar to the findings of Turgutalp et al. where the most common symptom among HD patients with COVID-19 was also difficulty of breathing [14].

Among the 3 groups, the average net fluid removal by week significantly differed, with the dialysis initiation group having the highest fluid removal, followed by the HD group, and lowest in the PD group. CKD patients may have had delayed consultation during the start of the pandemic because of an imposed lockdown; hence, with the dialysis initiation patients not on any form of renal replacement therapy, they came in the most congested, eventually needing the highest fluid removal. Between HD and PD, HD had higher fluid removal since it is an intermittent therapy, in contrast with PD where fluid can be removed daily as needed.

HD patients had significantly higher levels of inflammatory markers including LDH, hs-CRP, and procalcitonin compared to PD and dialysis initiation groups. High levels of inflammatory markers demonstrate the cytokine storm responsible for the increased severity of infection in COVID-19 patients, which may have resulted in an increased risk for mortality among HD patients compared to PD and dialysis initiation groups [4]. These results were similar to the study of Goicoechea et al. in Spain where increased levels of LDH and CRP were seen among nonsurvivors in their HD COVID-19 patients [6]. Findings were the same in a single-center study in New York by Valeri et al. where higher values of LDH and CRP were found among those who died [15]. Similarly, Jung et al. in their study in South Korea noted elevated CRP and procalcitonin among their 14 HD patients with COVID-19 [16]. In the multicenter study done by Turgutalp et al. in Turkey, HD patients all had elevated cytokine levels as well but, among them, it was elevated ferritin that was found to be a risk factor for mortality [14]. Conversely, in the study by Yiqiong et al. in Wuhan, there were reduced levels of inflammatory cytokines among HD patients [4]. It seems that the Chinese reports of lower mortality among dialysis patients are very different from other parts of the globe and the Philippine experience.

In general, about half of all patients exhibited multilobar pneumonia, with no significant difference among the 3 groups, suggesting a baseline radiologic pattern similar to that in the general population with COVID-19 [6].

In terms of immunomodulatory treatment, hemoperfusion was the most common treatment given and did not differ significantly among the 3 groups. This is because during the study period, protocols on COVID-19 treatment in the institute were still evolving, and hemoperfusion was available during this time. Hemoperfusion, an extracorporeal technique acting by adsorption mechanism, has been proposed as one of the treatment approaches to reduce inflammatory mediators involved in the cytokine storm occurring particularly in severe COVID-19 [17]. Two case reports have demonstrated improvement in terms of oxygen saturation and length of ICU stay after 3–4 sessions of hemoperfusion in severe COVID-19 [18, 19]. In a study done by Asgharpour et al. of 10 critically ill COVID-19 patients who received 3 sessions of hemoperfusion, 6 improved in terms of oxygen saturation, and in reduction in CRP and interleukin-6 [17]. Given the elevated inflammatory markers seen across all 3 groups, hemoperfusion showed great potential as adjunct treatment for severe COVID-19.

All patients were also given dexamethasone and anticoagulation using subcutaneous enoxaparin unless there were contraindications like bleeding. Remdesivir is contraindicated in patients with renal failure so it was not given to any patient. Convalescent plasma has to be procured from other hospitals so there was extreme difficulty in obtaining it due to competition from other hospitalized patients in the area.

In-hospital mortality and 30-day mortality were generally high for all CKD V patients, similar to the results of a multicenter study by Ng et al. in the USA where these patients had higher rate of in-hospital death compared to those without kidney disease [20]. Same findings were observed in European centers, and in a recent single-center US data [6, 7, 14, 15]. CKD V patients are believed to be in a proinflammatory state with functional defects in innate and adaptive immunity, predisposing to poor outcomes [21]. Moreover, these patients have other comorbidities like diabetes and hypertension, further increasing risk of death from COVID-19 [22].

Median time from COVID-19 confirmation to death was shorter at about a week for both HD and dialysis initiation groups, and longer at 2 weeks for the PD group. Consistently, in-hospital mortality, 30-day mortality, and probability of mortality were all significantly higher in both HD and dialysis initiation groups than the PD group but were not significantly different from one another. A reason for this could be that HD and dialysis initiation patients were more congested and uremic upon arrival, with no immediate means of fluid removal and clearance due to strict community quarantine measures during these months of the pandemic. In addition, during this time, it was difficult to find a hemodialysis center that catered to patients who tested positive for COVID-19 posthospital discharge; hence, some patients may have missed sessions, contributing to higher mortality. There were also no clear guidelines yet during this period on cohorting and attending to this set of patients in hemodialysis units.

In the HD group, the elevated inflammatory cytokines and higher mortality were also demonstrated in the studies that were done in Spain, Italy, UK, and India [6, 7, 23, 24]. In contrast, studies done in China and South Korea did not show poor survival for HD patients [4, 16]. Based on the results of this study, outcomes among HD patients in the Philippines are closer to Western countries than Asian ones. Interestingly, in the paper done by Kikuchi et al. in Japan, there was a high risk for overall mortality, though no significant difference was seen between HD and PD patients with COVID-19 [25]. However, their population was smaller at 100 patients (25 on PD and 75 on HD), and remdesivir was consistently given as treatment, which may have been a factor in survival. As previously stated, in this study, remdesivir was not given since it was not recommended for the dialysis population, which may have been partly contributory to the different outcome seen on mortality.

Meanwhile, the better outcomes for the PD group are consistent with the results of a multicenter study by Jiang et al. in China where overall mortality among these patients was low, and the PD population was not considered a high-risk population for COVID-19 [26]. A case series by Sachdeva et al. in the USA had similar findings wherein hospitalized PD patients with COVID-19 had a relatively mild course, with majority being discharged [27]. Since PD can be done at home, the continuous fluid removal, and preserved residual kidney function, may have played a role in the better outcomes for this set of patients [28]. Furthermore, PD being home-based resulted in less healthcare worker contact and less exposure to crowded environments, in contrast with HD and dialysis initiation patients who had regular exposure to healthcare workers and other patients in hemodialysis units. In the recommendations published by the Canadian Society of Nephrology in the year 2020, PD has been identified as the preferred modality for maintenance hemodialysis during the pandemic on the basis of its benefits likely outweighing risks [29]. Canney et al. described their PD experience in British Columbia between March 17 and June 1, 2020, in which a higher overall uptake of PD was noted compared to preceding years, with none of these patients started on PD being diagnosed with COVID-19 [30]. This PD experience in Canada further supports the better outcomes among PD patients amidst the COVID-19 pandemic, parallel with the findings in this study.

It is very clear from this study that HD patients and those requiring dialysis initiation are at higher risk for death; thus, strategies must be implemented to treat them more aggressively in terms of fluid removal. Daily HD treatments or alternating HD and iso-ultrafiltration for fluid removal during the early period of admission would be very helpful to improve their condition and may lead to improved survival. The significantly higher inflammatory markers among HD patients also warrant more aggressive management. The use of hemoperfusion in this population or other cytokine-directed therapies warrants further study in this high-risk population.

### 4.1. Recommendations and Limitations of the Study

Since this utilized a retrospective design, data gathering was limited to chart review. Residual renal function among the population and peritonitis rates among PD patients were not documented and, hence, were not reported in this study. Moreover, because it was done in a single center, outcomes depended on diagnostic and management protocols of the institute during its duration, which have evolved since then, and which may differ from those of other institutions locally and internationally. Furthermore, since there was no association component in this study, only comparison among the groups was done. Establishment of predictors of mortality among baseline variables was not performed.

Given more new information on COVID-19 particularly on management, further studies can be done determining an association between treatment received and mortality, while controlling for baseline variables.

## 5. Conclusion

In conclusion, among CKD V patients admitted for COVID-19 at the NKTI, those on HD and just initiating dialysis had higher in-hospital mortality and 30-day mortality, compared to patients on PD. HD and dialysis initiation patients need aggressive fluid removal early in their treatment and may improve with cytokine-directed therapies.

## Figures and Tables

**Table 1:** Baseline characteristics of CKD V patients with COVID-19 (*n* = 436).

	All patients (*n* = 436) *n* (%)	HD (*n* = 298) *n* (%)	PD (*n* = 103) *n* (%)	Dialysis initiation (*n* = 35) *n* (%)	*p* value
*Age (in years), median*	52.5 [IQR:40.5–60]	53 [IQR:41–61]	50 [IQR:33–60]	51 [IQR:43–63]	0.1147^a^
≥60 years	123(28)	86(29)	26(25)	11(31)	0.709^b^
<60 years	313(72)	212(71)	77(75)	24(69)	
*Sex*					
Male	245(56)	167(56)	57(55)	21(60)	0.887^b^
Female	191(44)	131(44)	46(45)	14(40)	
*Primary kidney disease, %yes*					
DM	146(33)	103(35)	36(35)	7(20)	0.211^b^
Hypertension	123(28)	89(30)	27(26)	7(20)	0.413^b^
CGN	129(30)	81(27)	35(34)	13(37)	0.254^b^
Others	40(9)	27(9)	5(5)	8(23)	0.006^*∗*^^b^
Charlson comorbidity index, median	4 [IQR:3–5]	4 [IQR:3–5]	4 [IQR:3–5]	4 [IQR:3–6]	0.5938^a^
*Chief complaint, %yes*					
Cough	35(8)	27(9)	5(5)	3(9)	0.397^b^
Fever	52(12)	41(14)	7(7)	4(11)	0.170^b^
Myalgia	32(7)	22(7)	6(6)	4(11)	0.546^b^
Headache	1(1)	1(1)	0	0	1.000^c^
Difficulty in breathing	187(43)	143(48)	33(32)	11(31)	0.007^*∗*^^b^
Sore throat	0	0	0	0	—
Diarrhoea	11(3)	5(2)	4(4)	2(6)	0.147^c^
Nausea	8(2)	5(2)	2(2)	1(3)	0.709^c^
Loss of smell/taste	51(12)	22(7)	24(23)	5(14)	<0.0001^*∗*^^b^
Others	214(49)	137(46)	58(56)	19(55)	0.158^b^
Dialysis duration (in years), median	1 [IQR:0.10–3]	1 [IQR:0.10–3]	0.90 [IQR:0.10–3]	—	0.0689^d^
Average net fluid removal per week (in ml), median	5376.5 [IQR:2947–7163]	5722 [IQR:3520–7310]	3790 [IQR:1892–5429]	6410 [IQR:1640–9330]	0.0001^*∗*^^a^

^a^Kruskal–Wallis test was used. Significant results were further analyzed using Dunn's test; ^b^chi-square test was used; ^c^Fisher's exact test was used; ^d^Mann–Whitney *U* test was used.

**Table 2:** Baseline laboratory and radiologic findings of CKD V patients with COVID-19 (*n* = 436).

	All patients (*n* = 436) Median [IQR]	HD (*n* = 297) Median [IQR]	PD (*n* = 103) Median [IQR]	Dialysis initiation (*n* = 35) Median [IQR]	*p* value
WBC count (in x10^9/L)	8.68 [IQR: 6–12.75]	8.45 [IQR:5.75–12.14]	8.94 [IQR:6.75–12.5]	11.23 [IQR:6.83–18.24]	0.0561^a^
Absolute lymphocyte count (in cells/microL)	948.68 [IQR:622.08–1277.82]	949.98 [IQR:609.03–1264.56]	930.95 [IQR:652.80–1302.84]	1002.50 [IQR:590.40–1317.84]	0.8442^a^
Hemoglobin (in g/dl)	9.2 [IQR:7.8–10.9]	9.2 [IQR:7.7–10.9]	8.9 [IQR:7.8–9.9]	10.85 [IQR:9.3–12.3]	0.0019^*∗*^^a^
Platelet (in 10 ^ 3/uL)	231 [IQR:168–305]	220 [IQR:156–278]	289 [IQR:206–373]	229 [IQR:179–329]	0.0001^*∗*^^a^
Sodium (in meq/L)	136 [IQR:132–140]	137 [IQR:134–141]	135 [IQR:131–140]	131.5 [IQR:127–135]	0.0001^*∗*^^a^
Potassium (in meq/L)	4.7 [IQR:4–5.7]	5 [IQR:4.2–5.9]	4.2 [IQR:3.5–5.1]	4.5 [IQR:3.8–5.2]	0.0001^*∗*^^a^
Corrected calcium (in mg/dl)	7.9 [IQR:7.2–8.5]	8 [IQR:7.3–8.6]	7.6 [IQR:6.9–8.4]	7.8 [IQR:7.2–8.6]	0.0067^*∗*^^a^
Albumin (in g/dl)	3.4 [IQR:3–3.8]	3.5 [IQR:3.1–3.9]	3 [IQR:2.35–3.45]	3.65 [IQR:2.8–4]	0.0001^*∗*^^a^
Ferritin (in ng/ml)	1253.60 [IQR:575.8–2322.4]	1275.35 [IQR:561.9–2381.2]	1189.15 [IQR:624.6–1824.6]	1339.1 [IQR:981.92–2369.65]	0.6630^a^
LDH (in IU/L)	367 [IQR:273–516]	391 [IQR:282.5–540]	344 [IQR:257–431]	315.5 [IQR:225.5–422.5]	0.0307^*∗*^^a^
hs-CRP (in mg/L)	78.60 [IQR:31.14]	88.9 [IQR:35.76–155.85]	55.61 [IQR:14.08–107]	77.38 [IQR:34.42–106.36]	0.0299^*∗*^^a^
Procalcitonin (in ng/ml)	1.93 [IQR:0.54–11.15]	3.26 [IQR:0.78–14.98]	0.75 [IQR:0.42–2.44]	1.57 [IQR:0.18–4.37]	0.0008^*∗*^^a^
Normal	61(14)	36(12)	16(16)	9(27)	0.097^b^
Single-lobe pneumonia	118(28)	75(26)	31(30)	12(36)	
Multilobar pneumonia	208(49)	153(52)	46(45)	9(27)	
Interstitial/diffuse bilateral pneumonia	41(10)	29(10)	9(9)	3(9)	

^a^Mann–Whitney *U* test was used; ^b^chi-square test was used.

**Table 3:** Treatment given to CKD V patients with COVID-19 (*n* = 436).

	All patients (*n* = 436) *n*(%)	HD (*n* = 298) *n*(%)	PD (*n* = 103) *n*(%)	Dialysis initiation (*n* = 35) *n*(%)	*p* value
Hydroxychloroquine	27(6)	20(7)	5(5)	2(6)	0.791^a^
Lopinavir/ritonavir	12(2)	11(4)	1(1)	0	0.203^a^
*Tocilizumab*					
1 dose	18(4)	15(5)	0	3(9)	0.033^*∗*^^a^
2 doses	2(1)	1(1)	1(1)	0	0.533^b^
Favipiravir	4(1)	2(1)	1(1)	1(3)	0.324^b^
Hemoperfusion	43(10)	35(12)	5(5)	3(9)	0.125^a^
Convalescent plasma	5(1)	4(1)	0	1(3)	0.292^b^

^a^Chi-square test was used; ^b^Fisher's exact test was used.

**Table 4:** Outcomes of CKD V patients with COVID-19 (*n* = 436).

	All patients (*n* = 436) *n*(%)	HD (*n* = 298) *n*(%)	PD (*n* = 103) *n*(%)	Dialysis initiation (*n* = 35) *n*(%)	*p* value
*In-hospital mortality*					
Yes	147(34)	113(38)	21(20)	13(37)	0.005^*∗*^^a^
No (recovered)	289(66)	185(62)	82(80)	22(63)	
*30-day mortality*					
Yes	119(27)	94(32)	16(16)	9(26)	0.005^*∗*^^a^
No	317(73)	204(68)	87(84)	26(74)	

^a^Chi-square test was used.

## Data Availability

The datasets generated and/or analyzed during the current study are available from the corresponding author on reasonable request.
